# Multivariate control charts based on Mahalanobis distance for bivariate quality monitoring in petrochemical and pharmaceutical analyses

**DOI:** 10.1007/s00216-026-06673-1

**Published:** 2026-07-25

**Authors:** Juliana Sasaki Ijiri, Pablo Vilar Gonzales, Felipe Rebello Lourenço, Elcio Cruz de Oliveira

**Affiliations:** 1 Programa de Pós-Graduação em Fármaco e Medicamentos, Departamento de Farmácia, Faculdade de Ciências Farmacêuticas, Universidade de São Paulo, São Paulo, Brazil; 2 Postgraduate Programme in Metrology, Pontifical Catholic University of Rio de Janeiro, Marquês de São Vicente Street, 225, Gávea, 22453-900 Rio de Janeiro, RJ Brazil; 3 Logistics, Operational Planning and Control, Measurement and Product Inventory Management, PETROBRAS S.A., Av. Henrique Valadares 28, Rio de Janeiro, 20231-030 Brazil

**Keywords:** Quality monitoring, Fuel analysis, Pharmaceutical assays, D^2^ statistic, Multivariate statistical process control, Resampling

## Abstract

This study evaluates the practical implementation of a covariance-adjusted monitoring procedure based on the squared Mahalanobis distance (D^2^) for routine bivariate quality control data. The approach was assessed using four small datasets generated in petrochemical and pharmaceutical laboratories, representing paired analytical measurements commonly monitored in routine practice. Rather than proposing a new multivariate statistical process control statistic, the study focuses on a transparent, spreadsheet-compatible implementation of D^2^-based monitoring and on the empirical characterization of control-threshold variability under small sample conditions. The D^2^ chart was compared with conventional univariate charts and with chi-square-based upper thresholds. In most datasets, both univariate and multivariate charts indicated stable process behavior with no action-level signals, reflecting the predominantly in-control nature of the available data. One ritonavir–lopinavir dataset provided an illustrative action-level exceedance, suggesting how a covariance-adjusted statistic may flag localized joint deviations; however, this single event should not be interpreted as definitive validation of fault-detection capability. A permutation-based resampling strategy was used to examine the variability of control thresholds and signal probabilities across alternative historical-monitoring partitions. Overall, the results support the feasibility, interpretability, and reproducibility of the proposed bivariate workflow as a complementary tool for routine analytical monitoring, while indicating that more rigorous validation with independent datasets containing known deviations is required before broader claims regarding fault-detection performance can be made.

**Graphical abstract:**

**Figure:**
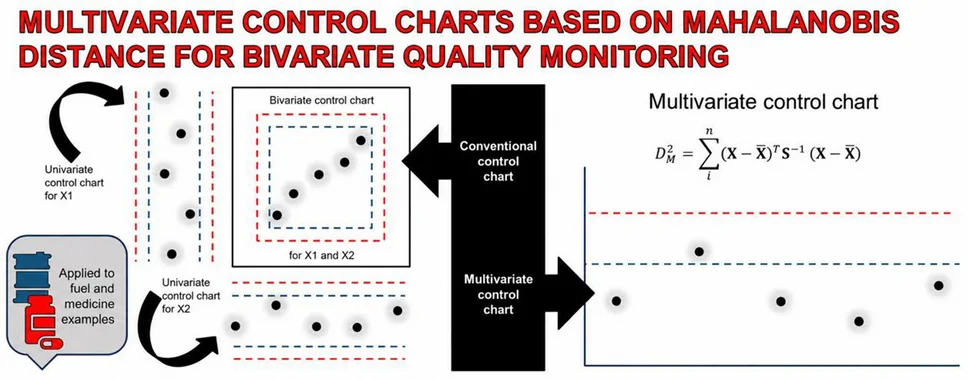

**Supplementary Information:**

The online version contains supplementary material available at https://doi.org/10.1007/s00216-026-06673-1.

## Introduction

Control charts, originally introduced by Shewhart and later disseminated through modern quality-management practice, remain central tools for distinguishing common-cause from special-cause variation in industrial and laboratory processes [1–4]. In the present context, the historical development of control charts is considered only as background; the main focus is their current role in analytical and bioanalytical quality control, where laboratories must monitor method performance, product quality attributes, and measurement stability under routine and regulatory constraints.

In analytical and bioanalytical laboratories, routine quality control tasks commonly involve method performance monitoring, verification of assay stability over time, comparison of related product attributes, and multi-attribute quality control. Examples include the joint monitoring of sulfur content with volatility-related fuel properties in fuels and the simultaneous monitoring of multiple active pharmaceutical ingredients in fixed-dose combination products. These applications require statistical tools that are simple enough for routine use but sufficiently informative to represent correlated analytical responses.

Recent examples further illustrate the continuing relevance of control-chart-based monitoring in pharmaceutical and industrial quality systems, including integrated quality-maintenance-production control in pharmaceutical processes, Shewhart-chart applications in pharmaceutical manufacturing inspection, distribution-free monitoring frameworks for operational processes, and empirical Bayes attribute control charts for quality monitoring [5–8]. These studies reinforce that control charts remain active tools for modern quality assurance, while also motivating simplified workflows adapted to routine analytical datasets.

Together, recent studies on multiparameter conformity assessment, AI- and chemometric-assisted monitoring, sensor-based process control, MSPC applications, and chemometric foundations reinforce the growing importance of multivariate strategies for analytical quality monitoring [9–18]. This literature supports the need for practical workflows capable of handling correlated analytical responses in routine laboratory settings.

A persistent limitation in routine laboratory practice is that control charts are often applied one variable at a time, even though analytical quality attributes may be correlated because of common sampling, sample preparation, instrumental, physicochemical, or process sources of variability. Established multivariate statistical process control (MSPC) approaches, including Hotelling’s T^2^ charts, PCA-based T^2^/Q-residual monitoring, and PLS-based MSPC, provide rigorous frameworks for monitoring correlated variables [19–22]. The present study therefore does not aim to introduce a new MSPC statistic, but to evaluate a transparent and spreadsheet-compatible implementation of covariance-adjusted D^2^ (where D denotes the Mahalanobis distance and D^2^ denotes its squared form) monitoring for routine bivariate analytical datasets.

In the bivariate setting considered here, the squared Mahalanobis distance is closely related to Hotelling’s T^2^, because both rely on the historical mean vector and covariance matrix to evaluate joint deviations. The contribution of this work is therefore implementation-oriented: it provides a low-dimensional workflow for routine laboratories, combines empirical and chi-square-based upper control thresholds, and uses permutation-based resampling to characterize control-threshold variability and signal behavior under small-sample conditions. This restricted scope is intended to support practical reproducibility and interpretation in routine bivariate monitoring, while complementing rather than replacing established MSPC methods.

Recent MSPC applications have increasingly combined spectroscopic measurements, sensor fusion, PCA, PLS, and other chemometric tools to monitor complex analytical and industrial processes, including fermentation, pharmaceutical continuous manufacturing, polymer production, fruit sorting, and membrane bioreactors. These studies demonstrate the relevance of MSPC for fault detection and real-time quality monitoring; however, many rely on model-intensive strategies, high-dimensional data streams, or process-specific sensor configurations. This distinction motivates the present study, which focuses on a low-dimensional, spreadsheet-compatible D^2^ monitoring workflow for routine petrochemical and pharmaceutical QC datasets, with empirical assessment of control-limit uncertainty under small-sample conditions.

## Materials and methods

### Overview of the proposed monitoring framework

The proposed procedure was designed as a practical multivariate monitoring framework for paired analytical measurements obtained under routine petrochemical and pharmaceutical quality control conditions. Four bivariate datasets were evaluated, each containing 30 lots. For each dataset, 20 lots were used as the historical reference subset for estimating location, dispersion, covariance structure, and control limits, whereas the remaining 10 lots were used as the monitoring subset for evaluating alert and action signals.

For each historical subset, univariate means and standard deviations were first calculated for each quality attribute. In parallel, the historical mean vector and sample covariance matrix were estimated for the paired variables. Conventional univariate control charts were constructed separately for each variable, whereas the multivariate chart was constructed by calculating the squared Mahalanobis distance (D^2^) of each monitored observation relative to the historical mean vector and covariance matrix. This allowed each lot to be represented by a single covariance-adjusted monitoring statistic.

The empirical alert and action limits for the D^2^ chart were calculated from the historical subset and compared with theoretical limits derived from the chi-square distribution with degrees of freedom equal to the number of monitored variables. Finally, a permutation-based resampling procedure was applied to evaluate the stability of the estimated limits and the detection performance of the univariate and multivariate charts over 10,000 alternative historical-monitoring partitions. Therefore, the “Materials and methods” section describes only the procedures applied in this work, while the broader MSPC literature is used in the Introduction to motivate the methodological gap addressed by the study.

### Data sources, measurement procedures, and data acquisition

The four datasets used in this study were obtained from routine analytical quality-control records generated in petrochemical and pharmaceutical laboratories. The petrochemical datasets were collected in a Brazilian oil and gas industry, which consisted of paired measurements of sulfur content, ASTM D7039 [23], and flash point, ASTM D93 [24]in diesel samples; and sulfur content, ASTM D5454 [25], and final boiling point, ASTM D86 [26], in gasoline samples. The pharmaceutical datasets consisted of paired assay results for lamivudine and zidovudine tablets, and ritonavir and lopinavir tablets determined by high performance liquid chromatography (HPLC). Each dataset comprised 30 production or analytical lots, organized in chronological order according to the routine sequence of laboratory testing.

All measurements corresponded to routine release or quality control analyses performed according to validated laboratory procedures applicable to each product class. For the petrochemical datasets, sulfur content, flash point, and final boiling point were determined using standard analytical procedures routinely adopted for fuel quality assessment. For the pharmaceutical datasets, active pharmaceutical ingredient contents were determined using validated assay procedures suitable for fixed-dose combination tablets. The present study did not modify the original analytical methods; instead, it used the reported analytical results as input data for evaluating the proposed monitoring framework.

The data acquisition process consisted of extracting paired numerical results from laboratory quality-control records and arranging them by lot number. No additional preprocessing, smoothing, outlier removal, or transformation was applied before constructing the control charts. The same measured values were used for all calculations, including estimation of univariate means and standard deviations, covariance matrices, Pearson correlation coefficients, D^2^ values, empirical upper control thresholds, chi-square-based thresholds, and permutation-based simulations. This direct use of the original measured values was intended to preserve traceability between the raw data, the spreadsheet calculations, and the reported results.

**Fig. 1:**
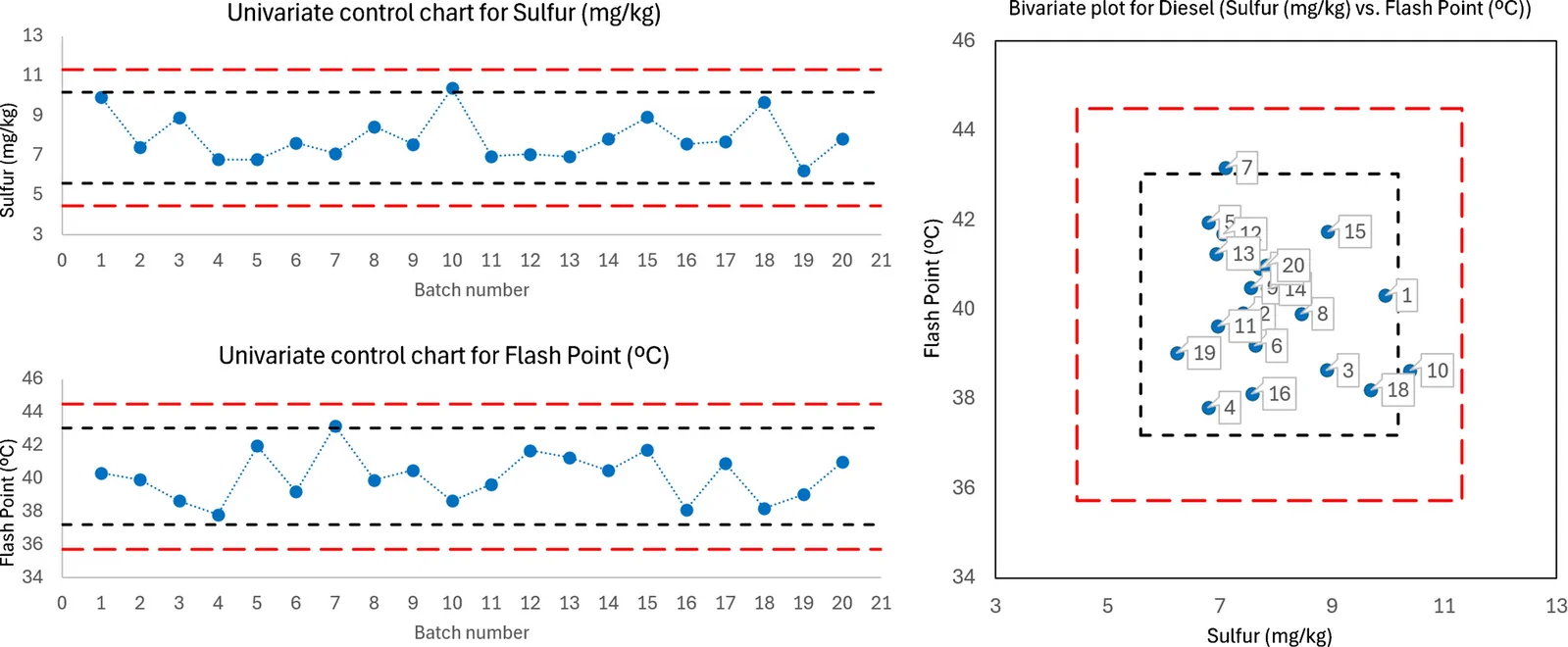
Univariate and bivariate control charts for sulfur (mg/kg) and flash point (°C) in Diesel analysis

**Fig. 2:**
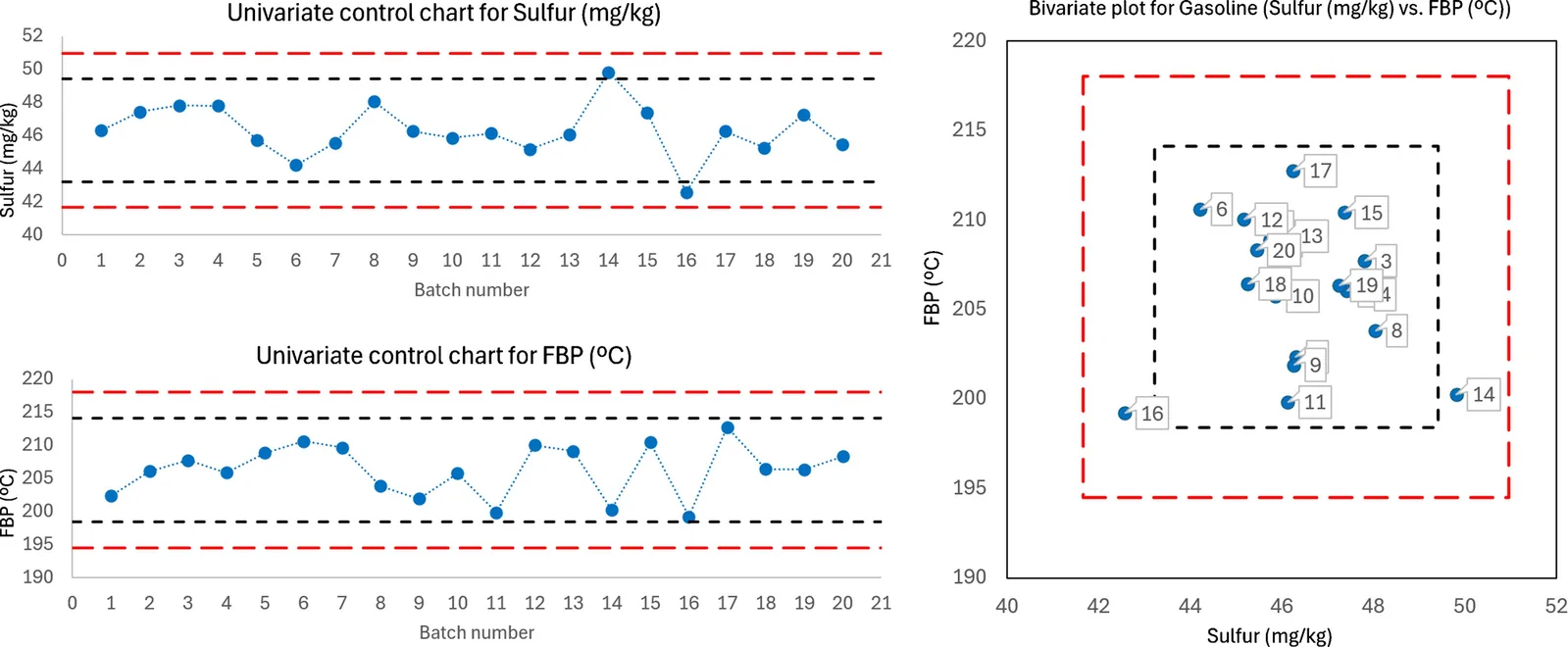
Univariate and bivariate control charts for sulfur (mg/kg) and FBP (°C) in Gasoline analysis

**Fig. 3:**
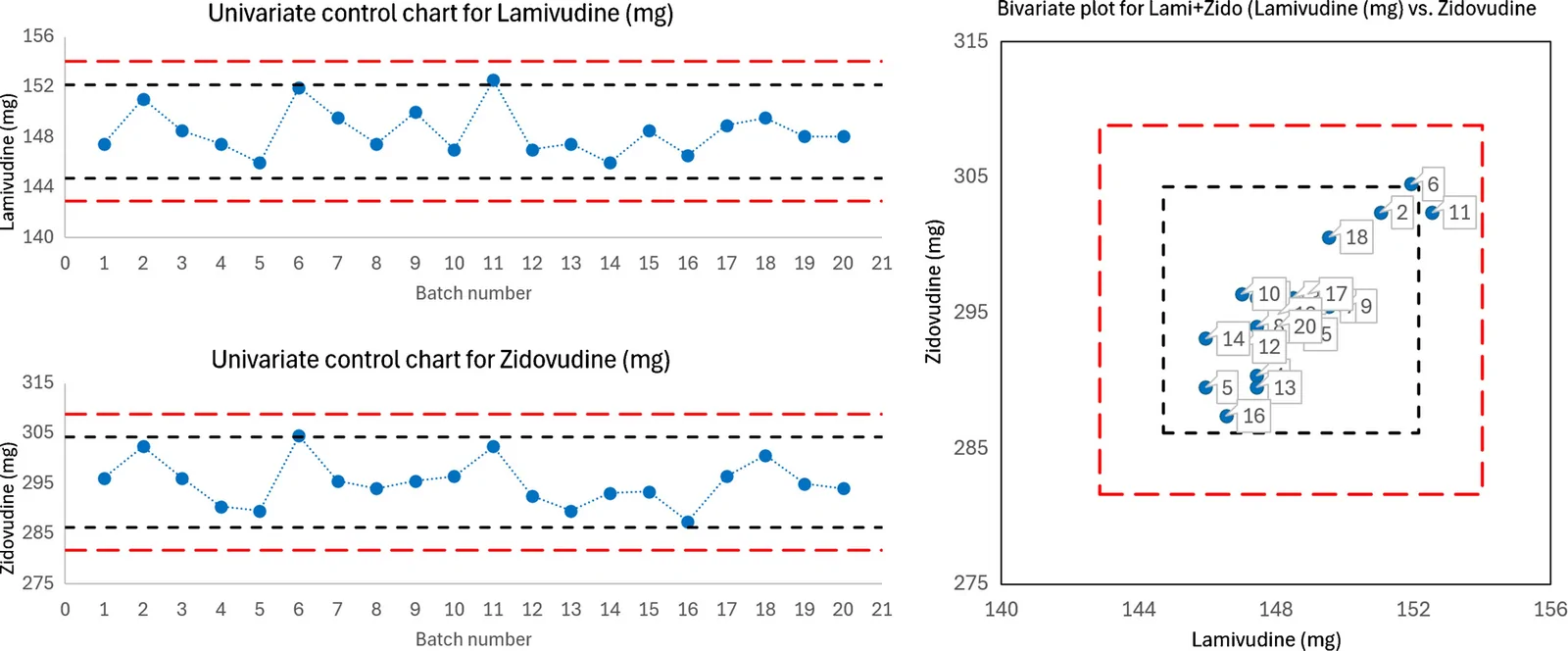
Univariate and bivariate control charts for Lamivudine (mg) and Zidovudine (mg) content in Lami + Zido tablets analysis

**Fig. 4:**
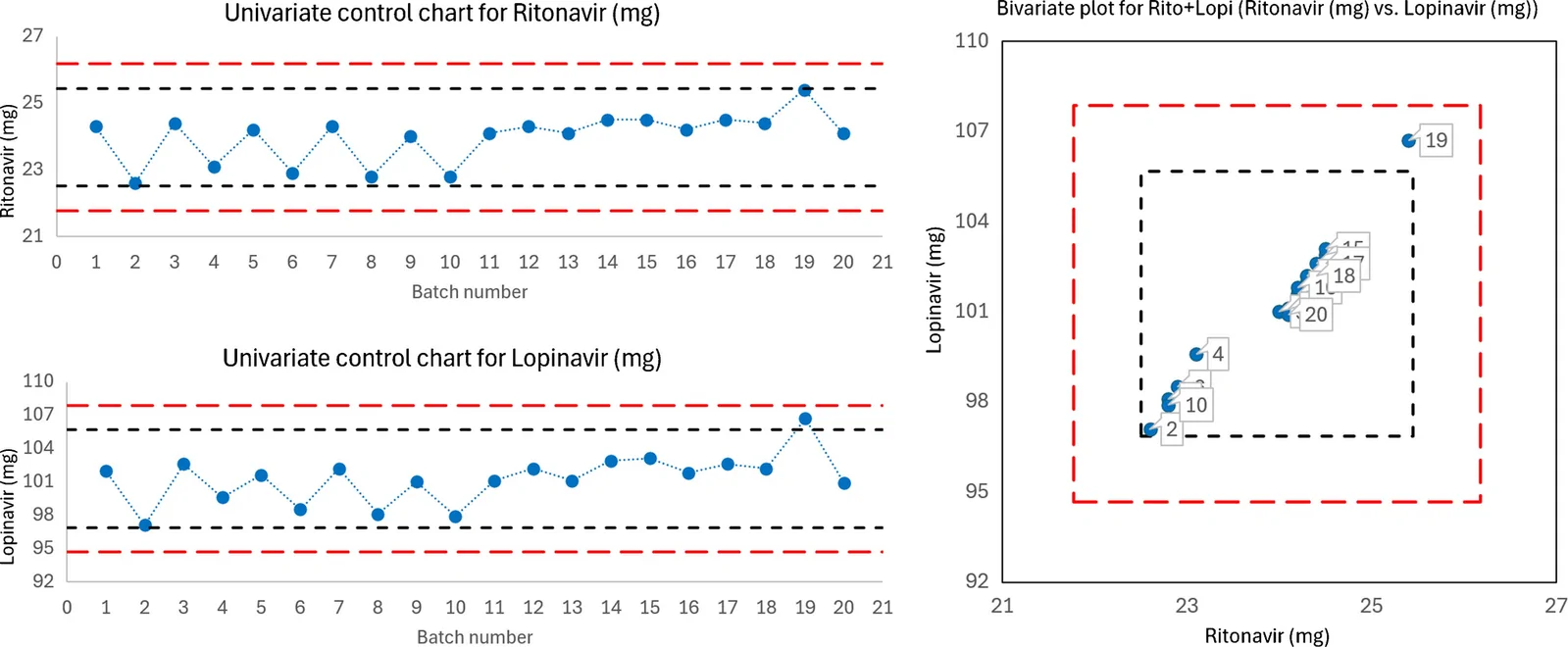
Univariate and bivariate control charts for Ritonavir (mg) and Lopinavir (mg) content in Rito + Lopi tablets analysis

**Fig. 5:**
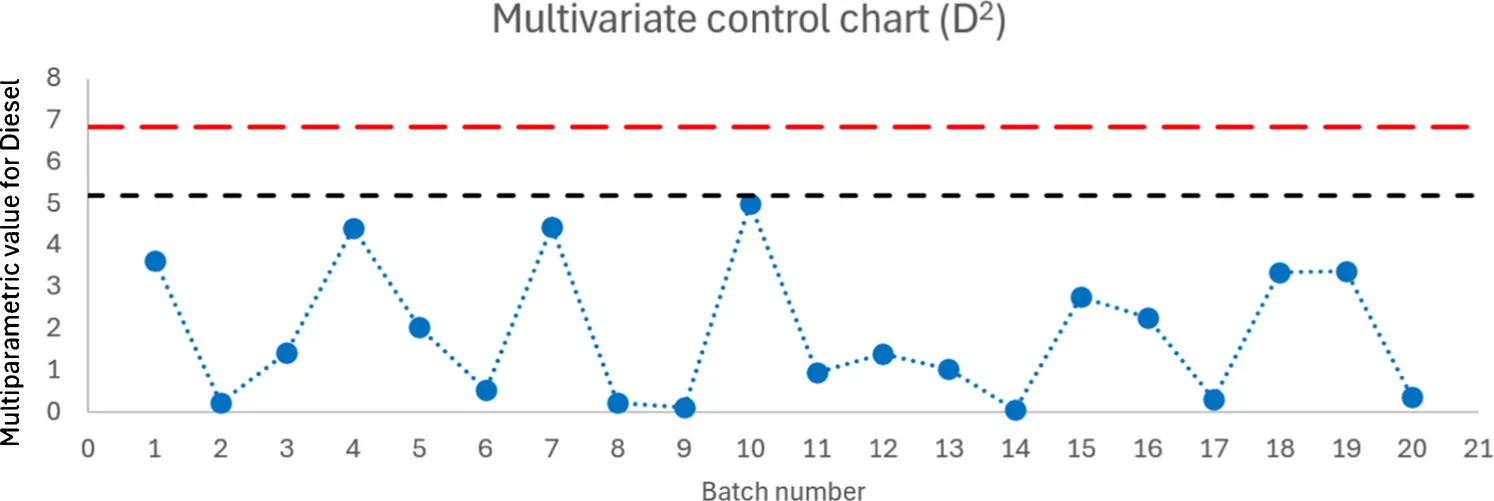
Multivariate (D^2^) control chart for sulfur (mg/kg) and flash point (°C) in Diesel analysis

**Fig. 6:**
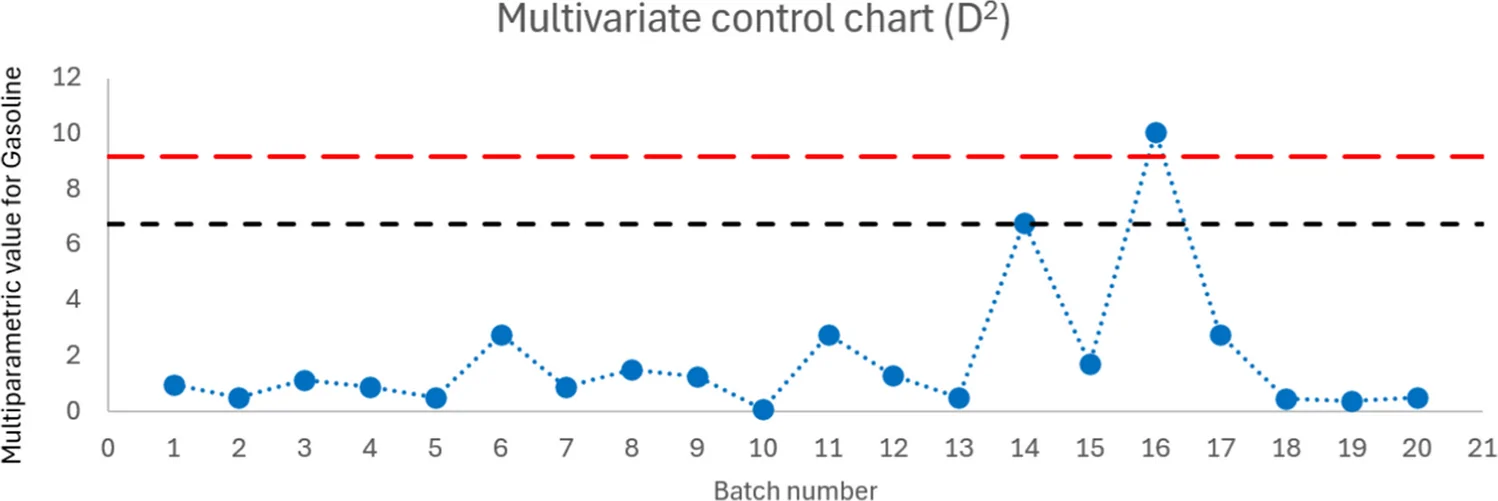
Multivariate (D^2^) control chart for sulfur (mg/kg) and FBP (°C) in Gasoline analysis

**Fig. 7:**
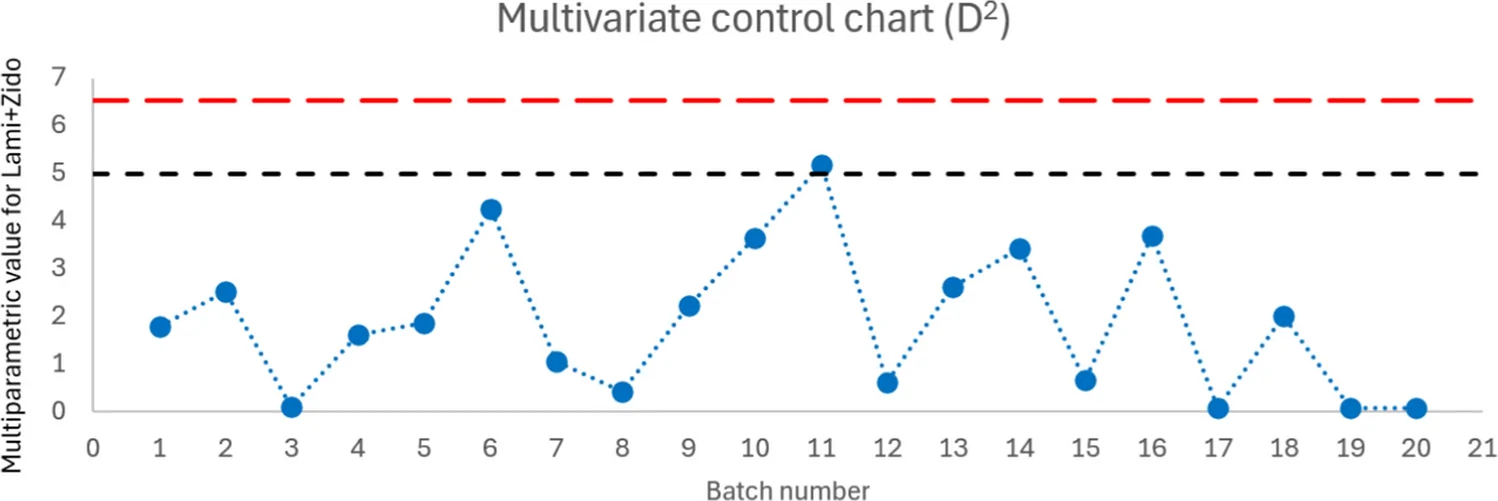
Multivariate (D^2^) control chart for Lamivudine (mg) and Zidovudine (mg) content in Lami + Zido tablets analysis

**Fig. 8:**
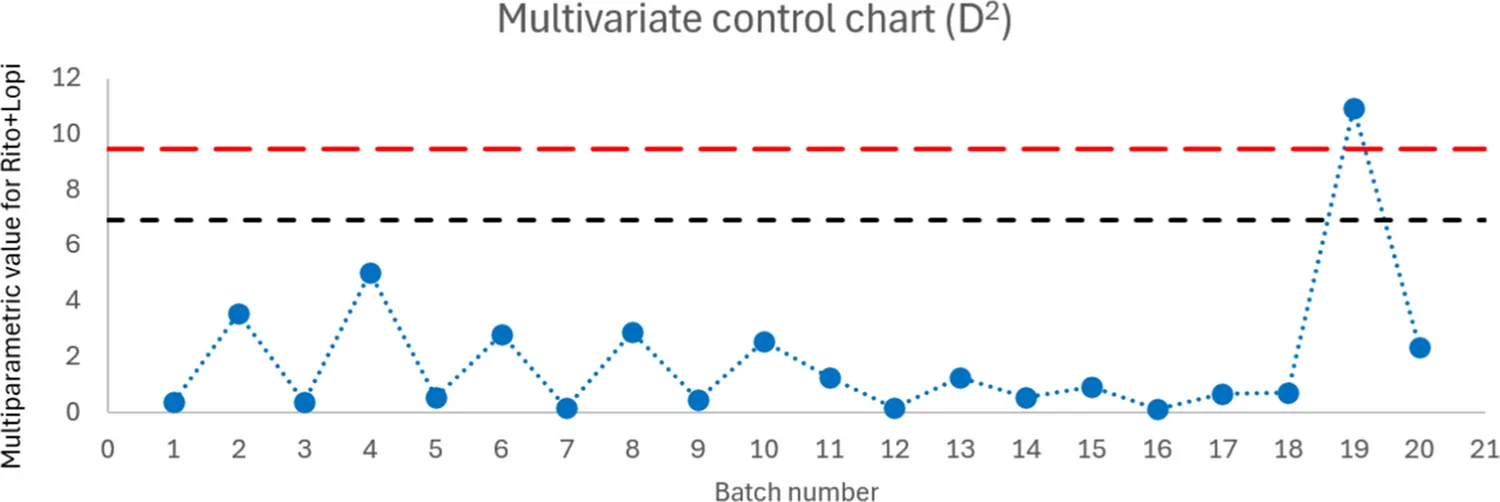
Multivariate (D^2^) control chart for Ritonavir (mg) and Lopinavir (mg) content in Rito + Lopi tablets analysis

The supplementary Excel spreadsheet was developed using Microsoft 365 Excel (Version 2606). It contains the raw measured values for the 30 lots of each dataset, the formulas used to calculate univariate means, standard deviations, covariance matrices, Pearson correlation coefficients, D^2^ values, empirical upper alert and action thresholds, chi-square-based thresholds, and permutation-based summary statistics. The spreadsheet also identifies which lots were assigned to the historical and monitoring subsets in the illustrative analysis and provides calculation sheets that allow readers to reproduce the values reported in Tables 1, 2, 3, and 4 and Figs. 1, 2, 3, 4, 5, 6, 7, and 8.

**Table 1:** Alert and action limits for univariate and bivariate control charts, as well as alert and action limits for multivariate control charts

Scenarios and parameters		Univariate and bivariate		Multivariate (D^2^)			
				Empirical		Chi-square	
		Alert limits	Action limits	Alert limit*	Action limit*	Alert limit**	Action limit**
(#1) Diesel	Sulfur (mg/kg)	5.6–10.2	4.4–11.3	5.2	6.8	6.0	9.2
	Flash point (°C)	37.2–43.0	35.7–44.5				
(#2) Gasoline	Sulfur (mg/kg)	43.2–49.4	41.7–51.0	6.8	9.2	6.0	9.2
	FBP (°C)	198.4–214.1	194.5–218.0				
(#3) Lami + Zido	Lami (mg)	144.7–152.2	142.9–154.0	5.0	6.5	6.0	9.2
	Zido (mg)	286.2–304.3	281.6–308.8				
(#4) Rito + Lopi	Rito (mg)	22.5–25.4	21.8–26.2	6.9	9.5	6.0	9.2
	Lopi (mg)	96.9–105.7	94.7–107.9				

Univariate alert and action limits; *multivariate alert and action limits, and **multivariate alert and action limits from chi-square distribution (for two variables)

**Table 2:** 95% confidence intervals obtained from 10,000 permutation-based simulation for alert and action limits for univariate and bivariate control charts, as well as alert and action limits for multivariate control charts

Scenarios and parameters		Univariate and bivariate		Multivariate (D^2^)	
		Alert limits	Action limits	Alert limit	Action limit
(#1) Diesel	Sulfur (mg/kg)	(5.0–5.8)^a^–(9.5–10.8)^b^	(3.5–4.1)^a^–(10.6–12.3)^b^	(4.3–5.7)^b^* and 6.0**	(5.5–7.6)^b^* and 9.2**
	Flash point (°C)	(37.2–38.3)^a^–(42.5–43.3)^b^	(35.7–37.1)^a^–(43.7–44.8)^b^		
(#2) Gasoline	Sulfur (mg/kg)	(42.3–43.6)^a^–(49.2–50.5)^b^	(40.3–42.0)^a^–(50.7–52.5)^b^	(4.9–6.4)^b^* and 6.0**	(6.3–8.7)^b^* and 9.2**
	FBP (°C)	(197.3–200.4)^a^–(212.8–215.4)^b^	(192.9–196.8)^a^–(216.4–219.7)^b^		
(#3) Lami + Zido	Lami (mg)	(144.0–144.9)^a^–(150.5–152.1)^b^	(142.0–143.4)^a^–(152.0–154.1)^b^	(4.7–6.3)^b^* and 6.0**	(6.2–8.5)^b^* and 9.2**
	Zido (mg)	(285.4–288.1)^a^–(300.3–304.3)^b^	(280.9–284.8)^a^–(303.4–308.9)^b^		
(#4) Rito + Lopi	Rito (mg)	(21.7–22.4)^a^–(25.3–25.8)^b^	(20.7–21.6)^a^–(26.1–26.8)^b^	(4.5–7.0)^b^* and 6.0**	(5.9–9.6)^b^* and 9.2**
	Lopi (mg)	(94.4–96.8)^a^–(104.9–107.0)^b^	(91.4–94.7)^a^–(107.0–110.0)^b^		

Univariate alert and action limits; *multivariate alert and action limits, and **multivariate alert and action limits from chi-square distribution (for two variables). ^a^95% confidence interval for lower limits and ^b^95% confidence interval for upper limits

**Table 3:** Detection rate among the 10 control lots, based on alert and action limits for univariate and bivariate control charts, as well as alert and action limits for multivariate control charts

Scenarios and parameters		Univariate and bivariate		Multivariate (D^2^)	
		Alert limits	Action limits	Alert limit	Action limit
(#1) Diesel	Sulfur (mg/kg)	0–30%	0–0%	0–48%* and 0–38%**	0–28%* and 0–18%**
	Flash point (°C)	0–20%	0–0%		
(#2) Gasoline	Sulfur (mg/kg)	0–10%	0–0%	0–30%* and 0–20%**	0–20%* and 0–18%**
	FBP (°C)	0–20%	0–0%		
(#3) Lami + Zido	Lami (mg)	0–20%	0–10%	0–20%* and 0–20%**	0–20%* and 0–18%**
	Zido (mg)	0–36%	0–8%		
(#4) Rito + Lopi	Rito (mg)	0–20%	0–0%	0–30%* and 0–28%**	0–28%* and 0–20%**
	Lopi (mg)	0–30%	0–0%		

Detection rate was calculated as the number of control lots outside alert or action limits, divided by the total number of control lots (10). Results were expressed as 95% confidence intervals, obtained from 10,000 permutation-based simulations

**Table 4:** Probability of observing at least one alert or action signal among the 10 control lots, based on alert and action limits for univariate and bivariate control charts, as well as alert and action limits for multivariate control charts

Scenarios and parameters		Univariate and bivariate		Multivariate (D^2^)	
		Alert limits	Action limits	Alert limit	Action limit
(#1) Diesel	Sulfur (mg/kg)	44.4%	0.5%	72.1%* and 47.2%**	33.1%* and 5.6%**
	Flash point (°C)	48.1%	0.2%		
(#2) Gasoline	Sulfur (mg/kg)	60.9%	1.0%	62.3%* and 62.0%**	49.3%* and 38.8%**
	FBP (°C)	45.1%	0.1%		
(#3) Lami + Zido	Lami (mg)	56.4%	19.6%	69.5%* and 65.2%**	48.1%* and 27.6%**
	Zido (mg)	52.6%	10.4%		
(#4) Rito + Lopi	Rito (mg)	20.4%	0.0%	62.9%* and 57.1%**	57.1%* and 54.0%**
	Lopi (mg)	58.6%	2.0%		

Probability of observing at least one alert or action signal was calculated as the number of permutation-based simulations in which at least one of the 10 monitored lots was outside the alert or action limits, divided by the total number of simulations (10,000)

Because the objective of this study was to evaluate the statistical monitoring framework rather than to validate the underlying analytical methods, detailed instrumental conditions, calibration models, and sample preparation protocols are not reproduced here. These procedures were assumed to be previously validated within the routine quality control environments from which the data were obtained. This limitation is acknowledged because the reliability of the monitoring framework depends on the reliability and traceability of the original analytical measurements.

### Euclidean and Mahalanobis distance

For each lot *i*, the paired analytical results were represented by an observation vector ${x}_{i}$ containing *p* measured quality attributes. The historical process center was represented by the mean vector ${\overline{x} }_{i}$, estimated by 20 historical lots. If the variables are considered independently and expressed on comparable scales, the Euclidean distance between ${x}_{i}$ and ${\overline{x} }_{i}$ provides a direct measure of geometric separation from the historical center. For p parameters, this distance may be calculated as Eq. (1):

1 $${D}_{E}^{2}=\sum\nolimits_{i}^{n}{\left({x}_{i}-{\overline{x} }_{i}\right)}^{2}$$

However, Euclidean distance does not account for differences in scale or for correlation between variables. In analytical quality monitoring, paired measurements may covary because of common sample preparation, instrumental conditions, physicochemical relationships, or shared process sources of variability. For this reason, a raw geometric distance may either overestimate or underestimate the abnormality of an observation. The Mahalanobis distance overcomes this limitation by weighing the deviation vector by the inverse of the historical covariance matrix S. The inverse covariance matrix, S^−1^, defines the metric used by the Mahalanobis distance by rescaling the deviation vector according to the variance of each variable and the covariance between variables. Thus, deviations along directions of high natural variability receive lower weight, whereas deviations along directions of low variability receive higher weight. This covariance-adjusted metric is widely used in chemometrics and multivariate quality monitoring to account for scale differences and correlation among variables [18]. Throughout this manuscript, the squared Mahalanobis distance is denoted consistently as D^2^. The squared Mahalanobis distance can be calculated as Eq. (2):

2 $${D}^{2}=\sum_{i}^{n}{\left({{\boldsymbol{X}}}_{{\boldsymbol{i}}}-\overline{{\boldsymbol{X}} }\right)}^{T}{\mathbf{S}}^{-1}\left({{\boldsymbol{X}}}_{{\boldsymbol{i}}}-\overline{{\boldsymbol{X}} }\right)$$

where $$\mathbf{X}=\left[\begin{array}{c}{x}_{i}\\ \vdots \\ {x}_{n}\end{array}\right]$$, $$\overline{\mathbf{X} }=\left[\begin{array}{c}{\overline{x} }_{i}\\ \vdots \\ {\overline{x} }_{n}\end{array}\right]$$, and $$\mathbf{S}=\left[\begin{array}{ccc}{s}_{ii}& \cdots & {s}_{in}\\ \vdots & \ddots & \vdots \\ {s}_{ni}& \cdots & {s}_{nn}\end{array}\right]$$.

This quadratic form is directly related to Hotelling’s T^2^ statistic, which is widely used in multivariate statistical process control to monitor correlated quality characteristics. In the present work, D^2^ is used as a practical covariance-adjusted monitoring statistic rather than as a newly proposed statistic. Geometrically, D^2^ defines ellipsoidal contours around the historical mean vector. Observations located along directions of high natural covariance may have moderate D^2^ values even if their raw Euclidean distance is large, whereas observations located along directions of low covariance may produce high D^2^ values even when each individual variable remains within its univariate limits. This property is the basis for detecting correlated moderate deviations that may remain unnoticed when separate univariate charts are interpreted independently.

Pearson correlation coefficients were calculated for each pair of monitored variables using the 30 available lots in each dataset. These coefficients were reported to characterize the dependence structure of each bivariate dataset before constructing and interpreting the D^2^ charts. This step is important because the squared Mahalanobis distance explicitly incorporates the covariance matrix; therefore, datasets with weak, moderate, or strong correlations may produce different D^2^ contours, control limits, and signal patterns even when the marginal univariate variability is similar.

For the bivariate case, the covariance matrix and the corresponding expanded expression for D^2^ can be derived directly from Eq. (2). Considering two variables ${x}_{1}$ and${x}_{2}$, with variances ${s}_{1}^{2}$ and ${s}_{2}^{2}$, standard deviations ${s}_{1}$ and ${s}_{2}$, and Pearson correlation coefficientr, the covariance matrix is given by Eq. (3), and substitution of its inverse into Eq. (2) leads to Eq. (4). Equation (4) makes explicit how the correlation coefficient modifies the contribution of each standardized deviation to D^2^.

3 $${\mathbf{S}}^{-1}=\frac{1}{{s}_{1}^{2}{s}_{2}^{2}\left(1-{r}^{2}\right)}\left[\begin{array}{cc}{s}_{2}^{2}& -r{s}_{1}^{2}{s}_{2}^{2}\\ -r{s}_{1}^{2}{s}_{2}^{2}& {s}_{1}^{2}\end{array}\right]$$

where ${s}_{1}^{2}\,{ \mathrm{a}\mathrm{n}\mathrm{d} \,s}_{2}^{2}$ are the variances; and r is the correlation coefficient.

4 $${D}_{M}^{2}=\frac{1}{1-{r}^{2}}\left[{\left(\frac{{x}_{1}-{\overline{x} }_{1}}{{s}_{1}}\right)}^{2}+{\left(\frac{{x}_{2}-{\overline{x} }_{2}}{{s}_{2}}\right)}^{2}-2r\left(\frac{{x}_{1}-{\overline{x} }_{1}}{{s}_{1}}\right)\left(\frac{{x}_{2}-{\overline{x} }_{2}}{{s}_{2}}\right)\right]$$

Equation (4) shows that the squared Mahalanobis distance is not merely the sum of two independent standardized squared deviations when the variables are correlated. The cross-product term involving r adjusts the distance according to the direction and strength of the correlation. When r= 0, this cross-product term disappears, and Eq. (4) reduces to Eq. (5).

Finally, when the two variables are independent and the covariance matrix is diagonal, the Mahalanobis distance reduces to the sum of squared standardized deviations. In this special case, D^2^ becomes equivalent to a standardized Euclidean distance, as shown in Eq. (5):

5 $${D}_{M}^{2}={\left(\frac{{x}_{1}-{\overline{x} }_{1}}{{s}_{1}}\right)}^{2}+{\left(\frac{{x}_{2}-{\overline{x} }_{2}}{{s}_{2}}\right)}^{2}={z}_{E}^{2}$$

### Multivariate control chart

For the multivariate control chart, the squared Mahalanobis D^2^ was calculated for each monitored lot using the historical mean vector and covariance matrix estimated from the 20 reference lots. The resulting D^2^ values were then plotted sequentially as a single monitoring statistic representing the joint behavior of the two analytical parameters. Thus, the chart summarizes both the magnitude and the direction of the deviation from the historical process center while explicitly incorporating the covariance between variables. The upper limit for the multivariate control chart can be calculated as Eq. (6):

Because D^2^ is a squared, non-negative statistic, only upper control thresholds were considered for the multivariate chart. Unlike conventional Shewhart limits for approximately normal variables, symmetric mean ± k standard deviation limits are not appropriate for D^2^, because its empirical distribution is bounded below by zero and typically right-skewed. Therefore, the multivariate monitoring rule was based exclusively on upper alert and upper action thresholds.

6 $${D}_{{L}_{U}}={\overline{D} }_{M}^{2}+3{s}_{{D}_{M}^{2}}$$

where $${\overline{D} }_{M}^{2}=\frac{\sum_{j}^{m}{{D}_{M}^{2}}_{j}}{m}$$ and $${s}_{{D}_{M}^{2}}=\sqrt{\frac{\sum_{j}^{m}{\left({{D}_{M}^{2}}_{j}-{\overline{D} }_{M}^{2}\right)}^{2}}{m-1}}$$.

In this study, two operational thresholds were considered for the D^2^ chart: an alert limit, used to indicate observations requiring attention or closer inspection, and an action limit, used to indicate observations requiring more formal investigation. These empirical limits were calculated from the historical D^2^ values and should be interpreted as data-driven operational thresholds rather than theoretically optimal universal limits. Their adequacy was evaluated as a *posteriori* through permutation-based uncertainty quantification and detection-performance analysis.

In the present implementation, empirical D^2^ thresholds were interpreted as upper-only operational thresholds. No lower D^2^ control limit was used, because values close to zero simply indicate observations located near the historical multivariate center and do not represent abnormal process behavior. When empirical mean-plus-k-standard deviation thresholds are used for D^2^, they should be regarded as pragmatic upper screening limits rather than as symmetric Shewhart limits. For applications involving highly noisy or strongly skewed D^2^ distributions, percentile-based limits or chi-square-based limits are more appropriate.

Under multivariate normality and adequate covariance estimation, D^2^ follows approximately a chi-square distribution with p degrees of freedom, where p is the number of monitored variables. Because this distribution is non-negative and asymmetric,chi-square-based limits naturally define only upper control thresholds. For this reason, chi-square limits were used in this study as a theoretically consistent benchmark for the empirical upper D^2 ^thresholds. However, because the present applications involve small historical subsets and routine analytical data that may not fully satisfy asymptotic assumptions, empirical and resampling-based upper thresholds were emphasized to characterize the uncertainty associated with control-limit estimation.

For comparison, conventional univariate control charts were constructed for each individual parameter by plotting the measured value of each lot as a function of the observation sequence. For each variable, lower and upper alert and action limits were calculated from the historical subset using the corresponding mean and standard deviation. These univariate charts provide variable-specific interpretation and were retained as complementary diagnostic tools, whereas the D^2^ chart provides an integrated assessment of joint variability. The lower and upper limits for the conventional control chart can be calculated as Eq. (7) and Eq. (8), respectively:

7 $${{L}_{L}}_{i}={\overline{x} }_{i}-3{s}_{{x}_{i}}$$

8 $${{L}_{U}}_{i}={\overline{x} }_{i}+3{s}_{{x}_{i}}$$

where $${\overline{x} }_{i}=\frac{\sum_{j}^{m}{{x}_{i}}_{j}}{m}$$ and $${s}_{{x}_{i}}=\sqrt{\frac{\sum_{j}^{m}{\left({{x}_{i}}_{j}-{{\overline{x} }_{i}}_{j}\right)}^{2}}{m-1}}$$.

### Permutation-based simulation

To assess the robustness of the proposed monitoring framework and to reduce dependence on a single historical-monitoring split, a permutation-based resampling strategy without replacement was implemented. For each of the four datasets, the 30 available lots were repeatedly reassigned into two subsets: 20 lots for historical estimation of means, standard deviations, covariance matrices, and control limits, and 10 lots for prospective monitoring.

The simulation algorithm was performed as follows. First, 10,000 random permutations of the 30 lots were generated without replacement. Second, for each permutation, 20 lots were assigned to the historical subset and 10 lots to the monitoring subset. Third, univariate alert and action limits were recalculated for each parameter from the historical subset. Fourth, the historical mean vector and covariance matrix were estimated, and D^2^ values were calculated for both historical and monitored lots. Fifth, empirical and chi-square-based D^2^ alert and action limits were obtained. Finally, the monitored lots were classified according to whether they exceeded alert or action thresholds in the univariate or multivariate charts.

For each simulated scenario, the following metrics were recorded: alert and action limits for all univariate charts; empirical and chi-square-based alert and action limits for the D^2^ chart; the number and proportion of monitored lots exceeding each threshold; and the probability of observing at least one alert or action signal among the 10 monitored lots. The empirical distributions obtained from the 10,000 scenarios were then used to calculate 95% confidence intervals for control limits and detection-performance metrics. This procedure provides a reproducible assessment of control-limit stability and monitoring sensitivity under small-sample conditions.

## Results and discussion

Multivariate control charts were obtained for four different scenarios: (#1) sulfur and flash point analyses in diesel; (#2) sulfur and final boiling point analyses in gasoline; (#3) lamivudine and zidovudine assays in tablets; and (#4) ritonavir and lopinavir assays in tablets. A summary of measured values obtained from 30 lots was presented in Table 5, and the corresponding Pearson correlation coefficients are reported in Table 6.

**Table 5:** Routine quality-control measured values obtained from 30 lots of diesel (#1), gasoline (#2), 3TC and AZT tablets (#3), and Ritonavir and Lopinavir tablets (#4)

	(#1) Diesel analyses		(#2) Gasoline analyses		(#3) 3TC and AZT tablets		(#4) Ritonavir and Lopinavir tablets	
Lot #	Sulfur (mg/kg)	Flash (°C)	Sulfur (mg/kg)	FBP (°C)	3TC (mg/tb)	AZT (mg/tb)	R (mg/tb)	L (mg/tb)
1	9.9	40.3	46.3	202.4	147.5	296.1	24.3	102.0
2	7.4	39.9	47.4	206.0	151.1	302.4	22.6	97.1
3	8.9	38.6	47.8	207.7	148.5	296.1	24.4	102.6
4	6.8	37.8	47.8	205.8	147.5	290.4	23.1	99.6
5	6.8	42.0	45.7	208.9	146.0	289.5	24.2	101.6
6	7.6	39.2	44.2	210.6	152.0	304.5	22.9	98.5
7	7.1	43.2	45.6	209.6	149.6	295.5	24.3	102.2
8	8.4	39.9	48.0	203.8	147.5	294.0	22.8	98.1
9	7.6	40.5	46.3	201.9	150.0	295.5	24.0	101.0
10	10.4	38.6	45.9	205.7	147.0	296.4	22.8	97.9
11	7.0	39.6	46.1	199.8	152.6	302.4	24.1	101.1
12	7.1	41.7	45.2	210.0	147.0	292.5	24.3	102.2
13	6.9	41.2	46.1	209.1	147.5	289.5	24.1	101.1
14	7.8	40.5	49.8	200.2	146.0	293.1	24.5	102.9
15	8.9	41.7	47.4	210.4	148.5	293.4	24.5	103.1
16	7.6	38.1	42.6	199.2	146.6	287.4	24.2	101.8
17	7.7	40.9	46.2	212.8	149.0	296.4	24.5	102.6
18	9.7	38.2	45.3	206.4	149.6	300.6	24.4	102.2
19	6.2	39.0	47.3	206.3	148.1	294.9	25.4	106.7
20	7.8	41.0	45.5	208.3	148.1	294.0	24.1	100.9
21	8.4	40.9	44.3	204.7	146.0	288.6	22.5	96.9
22	5.4	39.6	44.8	207.8	147.5	294.0	24.1	101.1
23	6.0	39.3	48.9	208.5	147.5	293.4	22.5	97.0
24	6.2	41.3	46.1	214.6	147.0	294.6	24.1	101.0
25	9.2	41.3	49.8	208.9	146.0	294.0	22.9	98.6
26	10.1	40.5	46.8	200.5	147.0	296.1	24.6	103.2
27	7.3	42.3	46.2	205.1	147.5	297.0	22.4	96.4
28	8.9	41.1	48.7	201.8	147.0	295.5	24.5	102.7
29	8.6	41.6	43.6	208.8	146.0	292.5	22.4	96.5
30	7.4	40.7	44.6	207.6	147.0	293.4	25.5	106.9

Legend: *FBP*, final boiling point; *3TC*, lamivudine; *AZT*, zidovudine; *R*, ritonavir; *L*, lopinavir

**Table 6:** Pearson correlation coefficients between paired variables in the four datasets

Dataset	Paired variables	Pearson correlation coefficient (*r*)	Correlation level
#1 Diesel	Sulfur vs. flash point	0.2084	Weak
#2 Gasoline	Sulfur vs. final boiling point	−0.1808	Weak
#3 Lamivudine + zidovudine	Lamivudine vs. zidovudine	0.5218	Moderate
#4 Ritonavir + lopinavir	Ritonavir vs. lopinavir	0.9918	Strong

The correlation coefficients confirm whether the four datasets represent weak, moderate, or strong dependence structures between paired variables. This information is relevant because the D^2^ statistic does not evaluate deviations independently in each coordinate; instead, it evaluates the position of each observation relative to the covariance-adjusted geometry of the historical dataset. When correlation is weak, the D^2^ contours approach axis-aligned ellipses and the chart behavior tends to resemble standardized univariate monitoring. In contrast, when correlation is moderate or strong, the D^2^ contours become more elongated and rotated, making the chart more sensitive to deviations that occur against the dominant correlation pattern and less sensitive to variations that follow the expected joint trend. Therefore, reporting the correlation coefficients is essential for interpreting why the multivariate chart behaves differently across the four datasets.

Conventional univariate and bivariate control charts were obtained using 20 lots for calculation of alert and action limits and calculated using the supplementary Excel spreadsheet, which includes the raw data, calculation formulas, covariance matrices, correlation coefficients, D^2^ values, empirical and chi-square-based thresholds, and permutation-based outputs needed to reproduce the reported tables and figures. These charts allow visualization of each parameter individually, as well as the joint distribution of the paired variables.

In the fuel datasets, similar patterns were observed for both diesel (Fig. 1) and gasoline (Fig. 2) analyses.

For diesel, the sulfur concentration and flash point values measured across the lots remained largely within the established alert and action limits for each variable, indicating stable process behavior with only small fluctuations around the central tendency. Likewise, in the gasoline dataset, sulfur content and final boiling point (FBP) measurements also remained within their respective univariate control limits, showing variability consistent with routine analytical measurements and no clear indication of special-cause variation. Although the univariate charts suggest that both processes were under statistical control, the bivariate representations reveal the potential relationships between the monitored parameters—sulfur and flash point in diesel, and sulfur and FBP in gasoline. These joint plots highlight how correlations between variables can influence the overall process variability and may reveal subtle patterns that are not evident when parameters are evaluated independently. Consequently, the combined interpretation of the two variables provides additional insight into process behavior and reinforces the importance of considering multivariate relationships when monitoring fuel quality parameters.

For the pharmaceutical datasets, univariate and bivariate control charts of lamivudine and zidovudine content in tablets and ritonavir and lopinavir content in tablets are presented in Figs. 3 and 4, respectively.

In both cases, the content values fluctuate within the expected analytical variability range and remain within the predefined control limits. These results indicate a generally stable analytical process and consistent product quality across the analyzed lots. However, the bivariate representations reveal the intrinsic correlation between the quantified active pharmaceutical ingredients (APIs), particularly in combination drug products where analytical results may share common sample preparation and chromatographic conditions. Such correlations highlight a potential limitation of conventional control charts, which monitor each variable independently and may fail to detect shifts that only become evident when the joint variability between variables is considered.

A summary of the alert and action limits calculated for both the conventional univariate control charts and the proposed multivariate monitoring approach is presented in Table 6. For each dataset, limits were established for the individual parameters as well as for the multivariate statistic based on the squared Mahalanobis distance (D^2^). The results show that the univariate limits define acceptable ranges for each variable separately, while the multivariate limits define acceptable ranges for the combined multivariate behavior of the system. Notably, the D^2^ alert and action limits provide a single metric capable of summarizing the combined variability of the two parameters, incorporating their covariance structure. This approach therefore offers a more comprehensive criterion for identifying abnormal observations that may not be detectable when variables are assessed individually.

The multivariate control charts based on the Mahalanobis distance (D^2^) for the same four datasets were also obtained using 20 lots for calculation of alert and action limits.

In the fuel datasets, the multivariate control charts based on the squared Mahalanobis distance (D^2^) indicate stable multivariate behavior for both diesel (Fig. 5) and gasoline analyses (Fig. 6), with the D^2^ values remaining below the action limit and only some observations lying close to the alert threshold, thus evidencing the absence of action-level violations.

For diesel, the calculated D^2^ values remain below the defined alert and action limits for most observations, indicating that the joint variability of sulfur and flash point measurements is consistent with the historical process behavior and confirming the stability already suggested by the univariate charts. Similarly, in the gasoline dataset, the D^2^ control chart shows that the combined variability of sulfur content and final boiling point measurements remains within the expected multivariate control limits. Although some individual observations exhibit moderate fluctuations in the univariate charts, the multivariate approach indicates that these variations remain consistent with the covariance structure of the process. Since the D^2^ statistic used here is closely related to Hotelling’s T^2^ in the bivariate case, the results should not be interpreted as evidence that the proposed chart is superior to established Hotelling-type MSPC charts. Rather, they illustrate a spreadsheet-compatible implementation of covariance-adjusted monitoring and provide an empirical evaluation of control-threshold variability under the specific small-sample conditions considered in this study.

The pharmaceutical datasets further demonstrate the usefulness of the multivariate approach. In the lamivudine and zidovudine dataset (Fig. 7), the D^2^ values remain well within the control limits, indicating that the joint assay variability of the two APIs is consistent across lots. Because both compounds are quantified using the same analytical method, a certain degree of correlation between their measured values is expected, and the multivariate chart effectively captures this relationship while providing a simplified monitoring metric.

A similar pattern is observed for the ritonavir and lopinavir dataset (Fig. 8), where the D^2^ chart shows that most observations remain below the action limit, with one lot exceeding this threshold. This single action-level exceedance should be interpreted only as an illustrative example of how the D^2^ statistic may flag a localized joint deviation, rather than as sufficient evidence to validate the general fault-detection performance of the proposed chart. By integrating the variability of both assay results into a single monitoring statistic, the multivariate control chart may provide complementary information for identifying unusual joint behavior; however, its broader detection capability must be interpreted cautiously in the absence of multiple confirmed out-of-control observations.

It is important to note that the four real datasets analyzed in this study mainly represent routine in-control analytical conditions. In most datasets, neither the univariate charts nor the multivariate D^2^ chart produced action-level signals, indicating stable process behavior. From a quality control perspective, this is a desirable outcome; however, it limits the ability of real case studies alone to demonstrate fault-detection capability. Therefore, the absence of frequent action-level violations should not be interpreted as evidence of limited method sensitivity, but rather because of the predominantly stable nature of the available routine datasets.

Permutation-based simulation provided additional insight into the robustness of the control limits and detection metrics. By repeatedly changing the historical-monitoring split, this procedure characterized how small-sample partitioning affects estimated thresholds and signal probabilities.

Across the 10,000 simulated scenarios, the estimated alert and action limits for both univariate and multivariate control charts exhibited relatively narrow variability, as reflected by their 95% confidence intervals provided in **b**. This indicates that the limits derived from the original dataset are reasonably stable despite the limited number of historical observations.

The comparison between empirical and chi-square-based D^2^ limits also addresses the concern that Shewhart-type symmetric limits may be inappropriate for a squared non-negative statistic. In all case studies, the empirical D^2^ alert and action thresholds remained positive; nevertheless, the use of lower D^2^ limits was avoided because such limits would have no practical interpretation. The relevant monitoring condition for D^2^ is exceedance of an upper threshold, not departure from a two-sided interval.

The permutation-based analysis was used to examine how often alert or action signals would arise under alternative historical-monitoring partitions of the same routine datasets. It therefore complements the single-partition analysis by quantifying control-limit stability and signal probability under realistic small-sample conditions. Nevertheless, because the resampling procedure is based on datasets that are mostly in control, it cannot fully replace validation using independent datasets containing known or intentionally induced faults.

The analysis of detection performance, summarized in Table 3, showed that the proportion of control lots exceeding alert or action limits varied depending on the dataset obtained during permutation resampling. When comparing the multivariate limits obtained by the proposed approach and those derived from the chi-square distribution, a substantial overlap of the 95% confidence intervals for detection rates was observed in most scenarios. This indicates that, despite differences in the estimated control limits, the resulting detection performance remains broadly comparable between the two approaches.

The probability of observing at least one alert or action signal among the 10 monitored lots, presented in Table 4, provides an additional and practically relevant measure of monitoring sensitivity. For multivariate control charts, the probabilities obtained using the proposed empirical limits and the chi-square-based limits were again largely consistent, with overlapping or very close values in most cases. This behavior is particularly evident for the alert limits, where the probabilities are nearly equivalent across all datasets. For action limits, some differences are observed, reflecting the previously noted divergence between empirical and chi-square limits; however, these differences remain moderate and do not fundamentally alter the interpretation of detection capability.

The multivariate D^2^ chart suggested the potential to flag joint deviations that may not be consistently identified by univariate charts, particularly when moderate but correlated changes occur simultaneously in both variables. Given the limited number of action-level deviations in the original datasets, this finding should be interpreted as evidence of complementary monitoring behavior rather than as definitive validation of fault-detection performance. The combined analysis of Tables 3 and 4 indicates that, although theoretical chi-square and empirical limits may differ, especially for action thresholds, their practical impact on signal probabilities remains broadly comparable within the uncertainty estimated by permutation resampling.

Taken together, the results support the feasibility and methodological consistency of the proposed workflow under routine petrochemical and pharmaceutical analytical conditions. However, because the datasets were predominantly stable, the results should not be interpreted as definitive evidence of fault-detection capability. More rigorous validation will require independent datasets containing known process shifts, controlled deviations, or simulated fault scenarios with predefined magnitude and correlation structure.

Compared with PCA- or PLS-based MSPC methods, the proposed approach requires minimal model calibration and is particularly suitable for low-dimensional routine analytical datasets.

In this sense, the distinctive contribution of the present workflow is not the replacement of Hotelling’s T^2^ or other established MSPC methods, but the translation of the same covariance-adjusted monitoring principle into a transparent, low-dimensional, and spreadsheet-compatible procedure for routine analytical quality control. Unlike many standard MSPC applications that rely on specialized software, high-dimensional sensor data, or model calibration through PCA/PLS, the proposed implementation focuses on small bivariate datasets commonly available in petrochemical and pharmaceutical laboratories. Its added value lies in combining D^2^-based monitoring with empirical and chi-square-based upper thresholds, permutation-based assessment of control-limit stability under small-sample conditions, and practical case studies involving fuel properties and fixed-dose pharmaceutical assays. Thus, the workflow is intended as an accessible complementary tool for routine QC environments rather than as a competing alternative to fully developed Hotelling’s T^2^ or chemometric MSPC frameworks.

From a practical analytical-laboratory perspective, this simplicity is relevant because many routine quality control tasks involve a small number of correlated measurements rather than high-dimensional sensor data. The proposed workflow may therefore be useful for monitoring method performance over successive analytical batches, following stability-study assay results in products containing more than one active ingredient, and supporting multi-attribute quality control in fuel and pharmaceutical testing. In these contexts, the D^2^ chart provides a compact way to evaluate whether the joint behavior of related analytical responses remains consistent with historical laboratory performance, while preserving the interpretability needed for routine decision-making by analysts and quality-control teams.

Although the present workflow was intentionally demonstrated using bivariate datasets, the methodology has clear potential to be extended to multivariate datasets containing more than two correlated quality attributes. This is because the D^2^ statistic is defined from a mean vector and a covariance matrix of dimension *p*, where *p* is the number of monitored variables; therefore, the same workflow can, in principle, be applied to three or more variables by estimating the corresponding *p*-dimensional historical mean vector and *p* × *p* covariance matrix. The operational sequence would remain essentially the same: selection of a historical subset, calculation of D^2^ values for monitored observations, definition of upper empirical or chi-square-based thresholds, and resampling-based assessment of limit stability. In practical applications, however, extension beyond two variables requires larger historical datasets, careful assessment of covariance matrix conditioning, and, when necessary, robust or regularized covariance estimators to avoid unstable limits when the number of variables becomes large relative to the number of available observations. Therefore, the proposed workflow is not restricted mathematically to bivariate datasets, but its reliable use with higher-dimensional data should be validated with appropriate multivariate case studies.

A direct numerical comparison with a classical Hotelling’s T^2^ chart was not the primary objective of this study, because in the bivariate setting the D^2^ statistic is expected to produce closely related monitoring behavior when the same historical mean vector and covariance matrix are used. Future work should include explicit benchmarking against standard Hotelling’s T^2^ implementations, PCA-based T^2^/Q-residual monitoring, PLS-based MSPC, and robust multivariate control charts using datasets with known shifts [19–22].

While the four case studies examined in this work represent typical small-sample situations encountered in routine petrochemical and pharmaceutical quality control, they also highlight an important limitation of the present validation strategy: the real datasets are predominantly stable and contain few action-level deviations. By means of a permutation-based resampling framework, many alternative monitoring scenarios are generated through repeated reassignment of observations into historical and monitoring subsets. This strategy expands the statistical characterization of the available data and allows empirical assessment of control-limit variability and signal probabilities under realistic small-sample conditions. However, because the resampling is constrained by the variability already present in the original datasets, it does not fully reproduce independent severe faults or controlled out-of-control scenarios. Therefore, the findings support the practical feasibility and interpretability of the proposed chart, while more rigorous validation of detection power requires datasets with known deviations.

## Conclusions

The results indicate that multivariate control charts based on the squared Mahalanobis distance can serve as a practical complementary tool to conventional univariate monitoring when correlated quality attributes are routinely measured in petrochemical and pharmaceutical analyses. By condensing joint variability into a single monitoring statistic while preserving covariance information, the proposed approach facilitates integrated visualization of process behavior under routine laboratory conditions. Overall, the study supports the feasibility, interpretability, and implementation simplicity of the workflow under mostly stable small-sample conditions.

The present contribution should therefore be interpreted as an implementation-oriented study, not as the introduction of new multivariate statistics. Its value lies in providing a transparent, spreadsheet-compatible workflow for routine analytical laboratories, while established MSPC tools such as Hotelling’s T^2^, PCA-based T^2^/Q-residual monitoring, and PLS-based approaches remain important benchmarks for future validation studies [19–22].

A limitation of this study is that the available real datasets were predominantly representative of stable routine analytical processes, with few action-level deviations. Consequently, future studies should include independent validation datasets containing known process shifts, spiked deviations, or controlled fault scenarios to quantify sensitivity, specificity, false-alarm rates, and detection power more rigorously.

The interpretation of the D^2^ chart depends on the correlation structure of the monitored variables; therefore, reporting and discussing correlation coefficients should be considered an essential step when applying this framework to routine bivariate quality monitoring.

Because D^2^ is non-negative and right-skewed, its use in control charts should rely on upper-only empirical, percentile-based, or chi-square-based thresholds rather than on symmetric Shewhart-type limits. Conventional two-sided Shewhart limits remain applicable to the original univariate measurements, whereas the multivariate D^2^ chart should be interpreted only through exceedance of upper alert and action thresholds.

The scope of the present study is intentionally limited to a bivariate, static, and small-sample monitoring framework, because this setting is frequently encountered in routine petrochemical and pharmaceutical quality control laboratories. Rather than proposing a general high-dimensional or adaptive MSPC solution, the contribution of this work is to provide a transparent, spreadsheet-compatible implementation of a D^2^-based chart, together with empirical uncertainty assessment through permutation-based resampling. This restricted scope should therefore be understood as a practical design choice aimed at improving accessibility and reproducibility in routine analytical contexts, not as an indication that the present study supports broad high-dimensional, adaptive, or real-time industrial implementation.

Future work should proceed incrementally from this restricted framework. The immediate next step is to validate the proposed chart using independent bivariate datasets containing known process shifts or controlled deviations. Subsequent studies may then examine extensions to three or more correlated variables, alternative covariance estimators, and adaptive limits. Broader developments such as integration with real-time digital platforms or economic performance assessment should be considered only after the method has been more extensively validated under controlled fault conditions. Accordingly, future perspectives were narrowed to avoid implying that the present study already supports high-dimensional, adaptive, or real-time industrial implementation.

## Supplementary Information

Below is the link to the electronic supplementary material.

Supplementary file1 (XLSX 58.9 KB)

## Data Availability

All data generated or analyzed during this study are included in this published article and its accompanying supplementary information files.
